# All-solid-state chip utilizing molecular imprinted polymer for erythromycin detection in milk samples: Printed circuit board-based potentiometric system

**DOI:** 10.1007/s00604-023-05959-w

**Published:** 2023-09-21

**Authors:** Mahmoud A. Tantawy, Ali M. Yehia, Heba T. Elbalkiny

**Affiliations:** 1 Pharmaceutical Analytical Chemistry Department, Faculty of Pharmacy, Cairo University, El-Kasr-El Aini St, Cairo, 11562 Egypt; 2 Chemistry Department, Faculty of Pharmacy, October 6 University, 6 October City, Giza, Egypt; 3 School of Life and Medical Sciences, University of Hertfordshire Hosted By Global Academic Foundation, New Capital, Garden City, Cairo, R5 New Egypt; 4 Analytical Chemistry Department, Faculty of Pharmacy, October University for Modern Sciences and Arts, 6th October City, 11787 Egypt

**Keywords:** Erythromycin, Milk analysis, Molecular imprinted polymer, Potentiometric sensor, Printed circuit board

## Abstract

Detection of erythromycin (ERY) residues in commercial milk samples is crucial for the safety assessment. Herein, a printed circuit board was patterned as a feasible miniaturized potentiometric sensor for ERY determination in dairy samples. The proposed chip design fits to a 3.5-mm female audio plug to facilitate the potential measurements of working electrode versus reference one in this all-solid-state system. The sensor utilizes molecular imprinted polymer (MIP) for the selective recognition of the studied drug in such challenging matrix. The electrode stability is achieved through the addition of poly (3,4-ethylenedioxythiophene) nano-dispersion on its surface. The proposed device detects down to 6.6 × 10^−8^ M ERY with a slope of 51 mV/decade in the 1 × 10^−7^–1 × 10^−3^ M range. The results display high accuracy (99.9% ± 2.6) with satisfactory relative standard deviation for repeatability (1.6%) and reproducibility (5.0%). The effect of common antibiotic classes, namely, amphenicols, beta-lactams, fluoroquinolones, sulfonamides, and tetracyclines, can be neglected as evidenced by their calculated binding capacities towards the proposed MIP. The calculated selectivity coefficients also show a good electrode performance in the presence of naturally present inorganic ions allowing its application to different milk samples.

**Graphical abstract:**

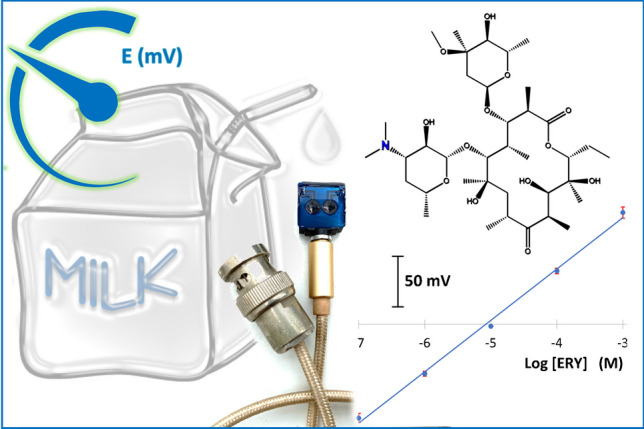

**Supplementary Information:**

The online version contains supplementary material available at 10.1007/s00604-023-05959-w.

## Introduction

Antibiotics are commonly utilized in veterinary medication for disease therapy and growth promotion in dairy cattle, but inappropriate antibiotic use and absence of monitoring after the withdrawal period in animals are resulting in the existence of their residues in human foodstuffs which are risky to human health [1]. Antibiotics in milky products can be controversial since they inhibit the growth of bacteria in the fermentation system causing some allergic reactions in hypersensitive individuals. Numerous studies state that taking low-dose antibiotics for long times may cause bacterial resistance which can be transmitted to humans, making certain infections challenging to be treated [2]. Erythromycin (ERY) is one of the most generally used members of macrolides class antibiotics. Belonging to a broad-spectrum class acting against gram-positive and some gram-negative bacteria, it is sometimes administered in drinking water or as an additive in cattle food to guard against certain diseases outbreak [3, 4].

Milk safety is receiving a lot of attention these days due to its global consumption and the harmful impact veterinary drug residues may generate on human. Regulation No. #2377/90 published by the European Commission, with its recent amendment demonstrated in rule No. #37/2010, states the maximum residue limits for certain antibiotics in milk, and it is 40 μg kg^−1^ for ERY [5]. Moreover, the banning of antibiotics use in veterinary medicine for growth promotion through feeding in livestock breeding has been announced by regulation No. #1831/2003 [6]. These firm limits demand the emergence of specific and sensitive analytical methods for antibiotic residue detection in milk. The two commonly reported techniques for ERY quantification in dairy samples include high-pressure liquid chromatography with Ms/Ms [4], electrochemical [7], or UV [8] detection and capillary electrophoresis with UV detection [9]. Chromatographic methods are cost and time prohibitive including multiple-step sample preparation as well as the significant low absorptivity of ERY that impedes UV detection signals [10]. Therefore, the development of an alternative electrochemical technique with satisfactory sensitivity and selectivity could be necessary for the fast determination of ERY in foodstuff samples. To this end, two feasible voltammetric methods have been developed for determination of ERY in dairy samples [11, 12]. However, no potentiometric method was reported for ERY monitoring in dairy samples till now.

Potentiometric methods are superior to voltammetric ones in terms of simplicity and analyte consumption [13]. Membrane potential is measured between two electrode systems for the detection of charged analytes. Conventional macro ion-selective electrodes (ISEs) need large sample volumes which pose a problem to limited sample and user-friendly applications. This problem could be solved through miniaturized electrochemical devices for pharmaceutical [14, 15] and biomedical [16] applications. Microelectrodes are widely considered one of the most significant advances in potentiometric devices. Several substrates can be employed in designing miniaturized sensors for commercialization [14, 16, 17]. On top of these materials, printed circuit board (PCB) technology is recently used to design standard two electrode systems on a small scale with high reliability and low cost [18]. This printed copper board is usually covered with different conducting materials for designing solid-state ISEs. MWCNTs are always preferred because of its relative hydrophobic nature that improves both the signal and electrode stability [19–22]. Nevertheless, water layer formation between the applied membrane and the conducting material may impede the ion-to-electron transfer between the ion-sensing membrane and the conducting solid surfaces. As a result, potential drifts arise by changing sample concentration since the slow re-equilibration of the accumulated electrolytes [23]. Under these circumstances, a hydrophobic conductive layer is incorporated at the membrane/electrode solid interface. It acts as a hydrophobic electron-transducer layer which makes aqueous layer formation unlikely. Poly(3,4-ethylenedioxythiophene) (PEDOT) is a widely used one due to its quite low band gap, superior properties such as air-stability, solubility in a variety of organic solvents, low ion contents, and high lipophilicity resulting in sensors with stable potential and low detection limit [14, 24–27]. It is also compatible with the common ionophores, and ion-exchangers used to prepare PVC sensing membranes.

Another important issue associated with potentiometry is the selectivity towards the analyte of interest where similar charged ions, as well as different matrix components such as proteins, lipids, and inorganic ions, may interfere [28]. One of the efforts that has been made to overcome those drawbacks is the introduction of molecular imprinted polymers (MIPs) in sensing membranes to specifically bind the required analyte [29, 30]. MIPs are polymers that have a high recognition capacity, long-term constancy, and good selectivity. They are built using the targeted drug as a molecular mold, and an efficacious monomer, via either covalent or non-covalent chemical bonds, accompanied by polymerization with the aid of a cross-linker to form an effectively linked polymer lattice. On eradication of the molecular template from the polymer’s network, definite recognition sites complementary to the template, in terms of their shape, size, and function, are uncovered to rebind to analyte molecules with high selectivity and affinity [31]. Analysts have recently exploited these unique selective characters of MIPs to enhance ISEs sensitivity and selectivity, especially in the case of complex sample matrices [32–36]. Being a complex matrix that is rich in proteins and lipids, milk analysis usually requires a careful pre-treatment to reduce those possible effects [37]. In addition, presence of various amounts of drug residues hinders direct assay of a certain analyte. As a result, molecular imprinting technology is developing rapidly in this field providing excellent materials for improved selectivity and sensitivity [11, 12, 38–40].

In this work, the dual advantage of potentiometric and MIPs technologies was exploited in designing the first PCB chip for the potentiometric assay of ERY in dairy samples. Engraved PCB was mounted to female audio plug to facilitate the connection to potentiometer. The chip contains reference and indicator multi-walled carbon nanotubes (MWCNTs) electrodes on the copper surface and incorporated with dispersed PEDOT nanoparticles as a conducting polymer to guard against formation of undesired water layer and advance electrode stability. In addition, the prepared MIP was incorporated into ion-sensing membrane to enhance its recognition affinity. This smart integration of miniaturization and molecular imprinting technologies enables the easy, fast, and selective detection of ERY from micro volumes of milk samples. Finally, the proposed all-solid-state potentiometric device was compared to other reported electrochemical sensors for ERY detection in different matrices.

## Experimental

### Materials and reagents

ERY (USP) Reference Standard with purity 99.5% (CAS No. 114078), ciprofloxacin HCl monohydrate (98.5%), tetracycline HCl (USP Reference Standard, 98.0% purity), azithromycin reference substance (98.2% of purity), methacrylic acid (MAA; 99%, with 250 ppm of MEHQ inhibitor), 2,2’-azobisisobutyronitrile (AIBN), ethylene glycol dimethacrylate (EGDMA; 98%, with 90–110 ppm of MEHQ inhibitor), tetrahydrofuran (THF ≥ 99.9%, anhydrous), dibutyl phthalate (DPB), potassium tetrakis[3,5-bis(trifluoromethyl)phenyl] borate (TFPB), tetradecyl ammonium bromide (TDAB), potassium chloride, nanoparticles water dispersion of poly(3,4-ethylenedioxythiophene) (PEDOT), acetonitrile (analytical grade), and dimethyl sulfoxide (DMSO), and multiwalled carbon nanotubes (MWCNTs, 10 ± 1 nm O.D × 4.5 ± 0.5 nm I.D × 36 μm L) were purchased from Sigma Aldrich, Germany.

A paste of MWCNTs (70% w/w) in mineral oil (Fluka, USA) was added to copper electrode surface. Polyvinyl chloride (PVC) of relatively high molecular mass purchased from Fluka, Germany. Milk products were purchased from a local Egyptian supermarket. Buffers of 3–10 pH range were prepared following Britton and Robinson method [41].

### Apparatus

Measurements were performed by the aid of Jenway pH meter 3505 (Jenway, UK). A high-resolution transmission electron microscope (HRTEM, Jeol-JEM2100, Japan) associated with the energy dispersive X-ray spectroscope (EDX) attachment. Fourier transform infrared spectrometer (FTIR, JASCO-460 plus, Japan). Field emission scanning electron microscope (FESEM, Fei Company-Quanta 250 FEG, USA). NOVAtouch (Quantachrome TouchWin™ version 1.2, Austria) for surface area and pore size analysis.

### Standard solutions

Stock solution of ERY (1 × 10^−3^ M) was prepared by accurately transferring 36.7 mg in a 50-mL volumetric flask. Around 20 mL of prepared buffer (pH 4.5) was introduced to dissolve, and then the volume was completed to mark using the same solvent. Different aliquots were diluted into 25-mL volumetric flasks to prepare working solutions with concentrations covering 1 × 10^−8^–1 × 10^−4^ M.

### Preparation of molecular imprinted (MIP) and non-imprinted (NIP) polymers

Polymers were synthesized via the bulk polymerization process according to reported method [42]. In a screw-plugged glass tube, 0.75 mmol (≈0.55 g) of ERY as a template drug was dissolved in DMSO pyrogen. This was followed by the addition of 3 mmol (≈0.26 g) MAA as functional monomer and shaking for 15 min to let the pre-polymerization composite to self-assemble. A total of 15 mmol (≈2.8 mL) of EGDMA as a cross-linker and 0.75 mmol (≈0.12 g) of AIBN as an initiator were then added. The solution was purged with argon for 5 min before being capped and retained in a water bath at 60 °C for 24 h under stirring. The polymer was collected and rinsed first with methanol/acetic acid (9:1, v/v) by the aid of Soxhlet extraction where complete extraction was monitored spectrophotometrically. After that, distilled water was applied successively to wash the formed MIP. Finally, the polymer was dried at 100°C for ≈ 1 h. The NIP was prepared and treated using the same method as for MIP, except that the template was not introduced.

Different physical and chemical characterization of MIP and NIP bulk materials were done. The physical characterization methods included (1) optical microscopy using HRTEM and FESEM for scanning MIP/NIP surface morphology, (2) EDX spectroscopy that helped in MIP elemental mapping, and (3) Brunauer–Emmett–Teller (BET) for the pore size and surface area determination. For the chemical characterization, FTIR spectra were recorded for MIP, before and after template removal, in the range of 500–4000 cm^−1^.

### Fabrication of all-solid-state sensor

For the reference membrane, 0.6 g solute mixture of 0.03 g TFPB and TDAB, 0.19 g PVC as well as 0.35 g DBP was dissolved in ≈ 4 mL THF. After that, 0.24 g AgCl and 0.64 g KCl, with silver traces, was added [43]. In case of PVC sensing membrane, it was fabricated by mixing 0.005 g of TFPB with 0.01 g of the prepared polymer (MIP or NIP) and 0.19 g PVC plasticized with 0.35 g DBP, and then the mix was dissolved in ≈ 4 mL THF. Forty microliters of 5 × 10^−2^ M aqueous ERY solution was added to avoid indicator membrane postconditioning [44]. The two membrane cocktails were sonicated for 15 min.

**Fig. 1:**
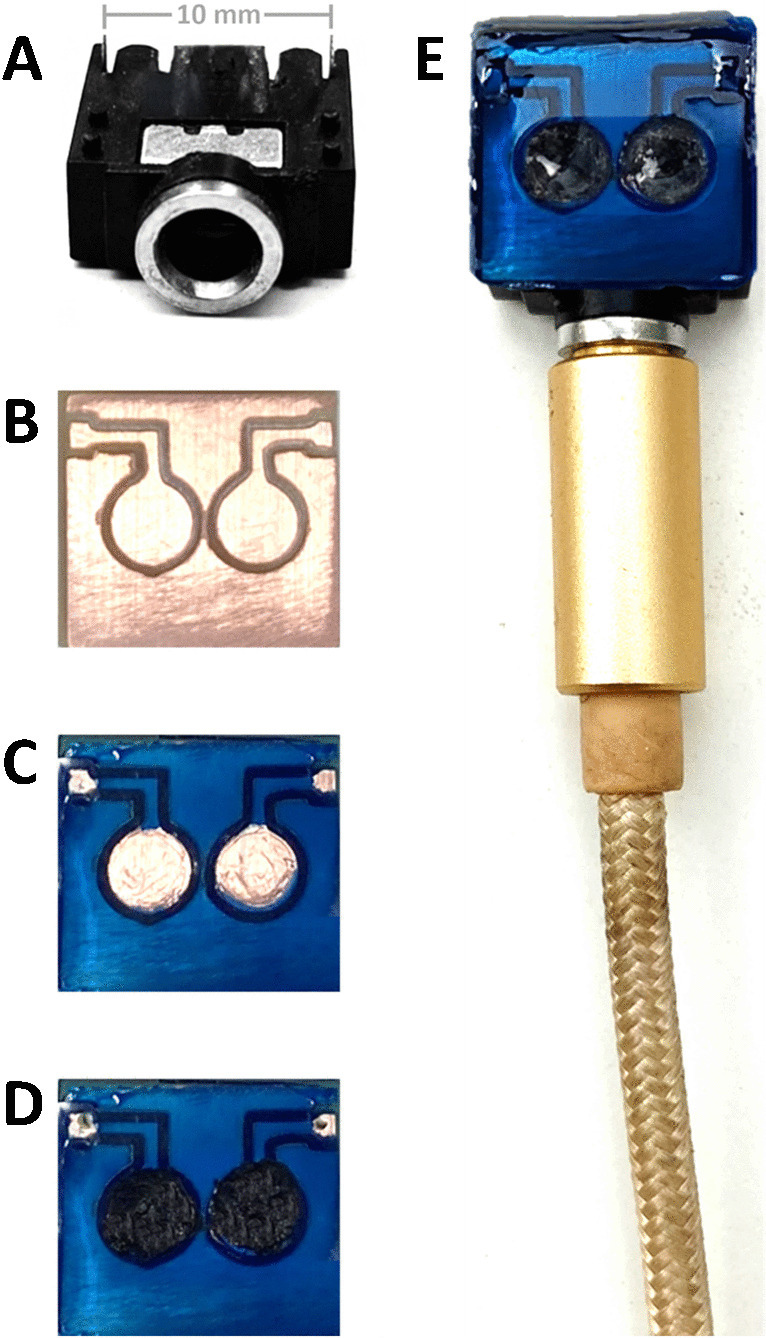
Female audio plug (3.5 mm) for printed circuit board mount (**A**). Printed circuit board after engraving (**B**) after masking with UV resin leaving exposed electrode areas and connection points (**C**) and after loading with MWCNTs at the electrode discs and drilling at the connection points (**D**). The final chip design mounted and soldered to the female audio plug (**E**). The chip was covered with sample hold sheet and loaded with sensing membranes at the electrode areas ready to be connected to potentiometer using male audio jack to male BNC cable

The all-solid-state device was designed in Inkscape vector graphics software to create scalable vector graphics (SVG) of the electrode chip. The designed chip dimension was 12 × 11 mm with electrical leads separated by 10 mm to fit 3.5-mm female audio plug at the connection points (Fig. 1A). Two circular discs with 13.6 mm^2^ area were also included as sensing electrodes. The design was then converted to 3D G-Code for milling using jscut (http://jscut.org/jscut.html#). Mini engraving machine connected to grbl Control 0.8 software to cut the PCB board in shape using 0.1 mm V-shape (30 degree) engraving carbide bit (Fig. 1B). UV mask was applied to the chip and cured under UV for 30 s while keeping the electrodes and connection areas masked. The uncured areas were wiped with ethanol to expose copper surface (Fig. 1C). About 5 mg of MWCNTs paste was applied on electrodes area to cover all copper surfaces, and then two holes were drilled into the PCB at the two connection points (Fig. 1D). The chip was mounted the female audio plug and the connection points were soldered and masked with UV resin. The whole chip surface was laminated with a transparent plastic film leaving an oblong hole around the circular electrode discs to hold the sample in the final design (Fig. 1E). Ten microliters of PEDOT was applied to each electrode followed by three successive additions of 5 µL of the membrane cocktails on their respective discs. The sample well was filled with 3 M KCl when the device was not in use. The chip is connected to potentiometer using 3.5-mm male audio jack to BNC plug.

### Potentiometric measurements

Dilutions from stock ERY solution (1 × 10^−3^ M) were performed in the range of (1 × 10^−8^–1 × 10^−3^ M) using a buffer of pH 4.5 as a solvent. Calibration was built through the successive introduction of 50 µL from those standard solutions in the defined sample well. Potential difference was measured between working and reference electrodes for each concentration. IUPAC recommendations were obeyed to evaluate performance characteristics of the proposed device [45, 46]. Water layer test [47] was carried out by immersing the electrode in 1 × 10^−2^ M solution of azithromycin after soaking in 1 × 10^−4^ M ERY then returning back to ERY solution. The influence of pH was also investigated using two concentrations (1 × 10^−5^ and 1 × 10^−4^ M) over 3–10 pH range at one pH unit interval. After that, selectivity coefficients of the proposed device towards different species that may be present in milk were calculated using separated solution method [48].

### Milk samples pretreatment and analysis

In 10-mL glass centrifuge tubes, aliquots of 2 mL from each spiked milk sample were treated with 1 mL acetonitrile to precipitate the milk proteins. The obtained supernatants were refined through 0.45-μm filters to ensure the removal of proteins and transferred to 10-mL measuring flasks. The potentials were then recorded after completing the volumes with a pH 4.5-buffer solution.

## Results and discussion

This study presents a simple, economic, and easy-to-use potentiometric approach for the detection of ERY in milk samples. The proposed PCB platform offers a cost-effective solution for upscaling and standardization of ERY electrochemical sensor. The maximum residue limit value of ERY (≈ 5.62 × 10^−8^ M) in milk requires the use of PEDOT as a conducting polymer to circumvent the water insulating layer for a stable potential and higher sensitivity. The complexity of milk samples and the possible presence of various lipophilic drug residues necessitate the incorporation of MIP as a recognition element that would improve the sensor selectivity. All those challenges were carefully studied and discussed below for designing ERY device with extraordinary analytical figures of merit.

### MIP characterization

In this work, the proportion of the chosen monomer (MAA) to the template (ERY) was set at 4:1 as per the previously reported procedure [11]. Moreover, DMSO was selected as an aprotic solvent since it is considered nontoxic and relatively stable at elevated temperatures [49]. The imprinting process and morphology characterization as well as pore size analysis of the prepared MIP were conducted by FTIR, HRTEM, EDX, FESEM, and BET/Barrett-Joyner-Halenda (BJH) techniques. UV-spectrophotometry was also applied to assess its rebinding competency.

The IR spectra of unleached and leached MIP were examined (Fig. S1, supplementary information). C-N and C = O stretching at 1168 and 1731 cm^−1^, respectively, along with the alcoholic OH band at 3356 cm^−1^ were shown at the unleached MIP spectrum. All those bands are characteristic of ERY drug, thus confirming the good imprinting process. On the other hand, the successful removal of the template drug was confirmed via the leached MIP spectrum where disappearance of those characteristic bands was observed. It is worth noting that both spectra were conserving the stretching vibrations of MAA.

**Fig. 2:**
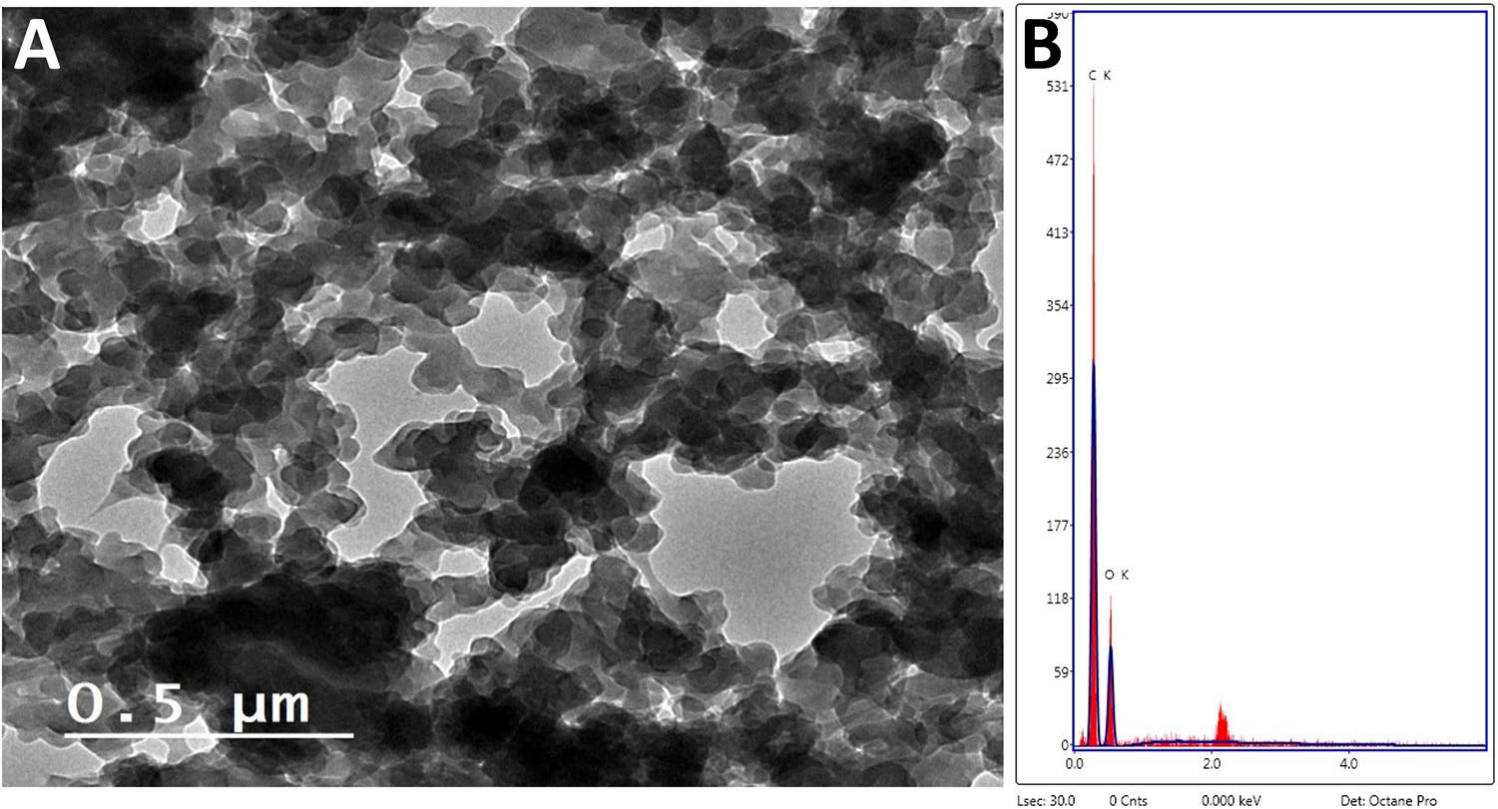
High-resolution transmission electron microscope image (**A**) and energy dispersive X-ray spectrum (**B**) of the prepared molecular imprinted polymer for erythromycin

The HRTEM image of leached MIP confirmed its irregular morphology associated with bulk polymerization process (Fig. 2A). In addition, the recorded EDX spectrum showed high percentages of carbon and oxygen atoms coming from functional MAA monomer (Fig. 2B).

**Fig. 3:**
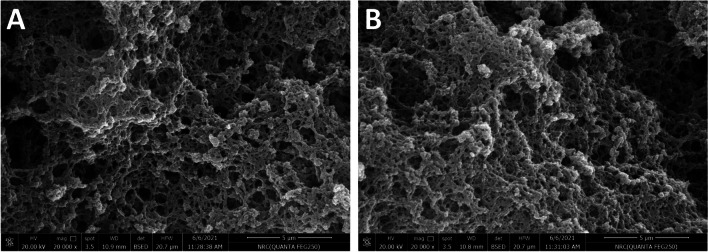
SEM images of the molecular imprinted polymer (**A**) and non-imprinted polymer (**B**) at 2 × 10^4^ × magnification power

The surfaces’ morphologies of leached MIP as well as its parallel NIP were studied via FESEM analysis (Fig. 3). Similar irregularities associated with bulk polymerization process were observed. However, more rough structure appeared for MIP image relative to the NIP one which may be attributed to the small cracks created after ERY removal.

BET and BJH tools were employed for calculations of surface areas and pore diameters, respectively. Before measurements, leached MIP and NIP samples were degassed at 60 °C under vacuum for 8 h to clean the polymers from adsorbed moisture and gases. After that, measurements were conducted at 77.35 K using nitrogen as an adsorbate. The results revealed higher surface area and pore volume for MIP; thus, effective imprinting is assured. Moreover, the gained pore diameters’ values demonstrated that the two substances are mesopores [50] (Table S1, supplementary information).

Finally, a simple UV-spectrophotometric technique was exploited to assess the rebinding competency of the prepared polymers, MIP and NIP, towards ERY. Twenty-five milligrams of MIP and its equivalent NIP was independently added to 5 mL 0.07 mM ERY solution in buffer pH 4.5. The suspensions were stirred, and then left for 2 h at ambient temperature. They were subsequently centrifuged for 15 min at 4000 rpm, and the obtained supernatants were filtered through 0.45 µm. UV absorbance was recorded at 285 nm, and free ERY concentration was calculated. The binding capacities (Q), in µmole g^−1^, were calculated for MIP and NIP based on this equation [36, 42]:

$$Q=\frac{\left({C}_{i}-{C}_{f}\right)\times V\times 1000}{{M}_{polymer}}$$

where *C*_i_ is the initial and *C*_f_ is the final free ERY concentrations in mM, *V* is the volume of solution in mL, and *M*_polymer_ is the polymer’s mass in mg (Table S1, supplementary information). The imprinted factor (IF) could be then deduced using the following equation:

$$\text{IF = }\frac{{\text{Q}}_{\text{MIP}}}{{\text{Q}}_{\text{NIP}}}$$

The IF of 3.4 was obtained indicating the superiority of MIP in the selective binding of ERY drug over the NIP. Furthermore, the MIP’s selectivity was assessed through calculating Q values after incubating the polymer with common antibiotics classes found in milk, namely, amphenicols, beta-lactams, fluoroquinolones, sulfonamides, tetracyclines, and structurally analogue azithromycin [51]. The before-mentioned procedures were followed, and the respective Q values were about 3–5 times lower than ERY one (Table 1). The proposed MIP could distinguish ERY from other common antibiotic residues that possibly excreted in milk.

**Table 1:** Calculated Q values of the fabricated MIP towards different antibiotics classes

Class	Antibiotic	Q (µmol g^−1^)^a^
Amphenicols	Chloramphenicol	2.1 ± 0.2
Beta-lactams	Amoxicillin	2.8 ± 0.1
	Cloxacillin	3.2 ± 0.2
Fluoroquinolones	Ciprofloxacin	3.5 ± 0.3
	Levofloxacin	3.1 ± 0.2
Sulfonamides	Sulfadiazine	2.7 ± 0.4
	Sulfathiazole	2.4 ± 0.3
Tetracyclines	Tetracycline	3.9 ± 0.2
	Doxycycline	3.3 ± 0.1
Structurally analogue	Azithromycin	4.2 ± 0.2

^a^Mean ± SD of three determinations

### Electrochemical characteristics of ERY device

**Fig. 4:**
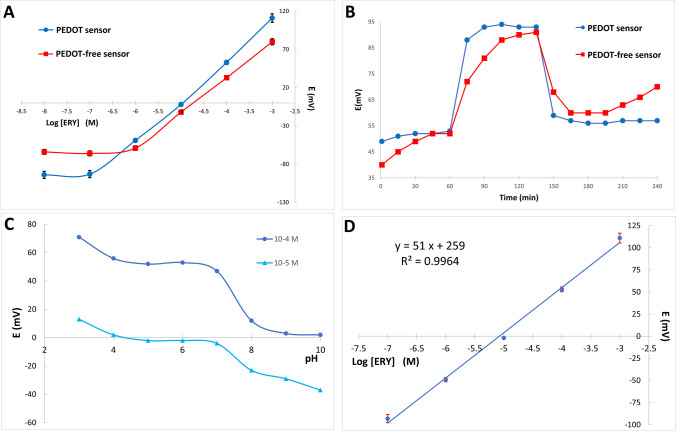
Potential profiles as a function of ERY concentrations using the proposed PEDOT sensor and PEDOT-free one (**A**). Water layer test recorded in 1 × 10^−4^ M ERY and 1 × 10^−2^ M azithromycin as interferent for PEDOT-based and PEDOT-free sensors (**B**). Effect of pH change on the potential response of the proposed sensor (**C**). The measure potential versus Log concentrations of ERY (1 × 10^−7^–1 × 10^−^.^3^ M) using the proposed sensor (**D**)

The presence of a basic tertiary amine group, in ERY structure (Fig. S2, supplementary information), suggested the use of TFPB, a highly lipophilic cationic exchanger, in the sensing membrane. It is physically compatible with polymeric matrix. Plasticizer is also essential for construction of PVC sensing membrane as it adjusts the membrane permittivity and allows ionic species to freely flow throughout the membrane matrix, hence improves membrane conductivity providing the highest possible selectivity and sensitivity [52, 53]. The use of DBP as a plasticizer in this study helped in attaining the best Nernstian slope for monovalent cations. This could be attributed to its high dielectric constant and capability to increase membrane polarity. PEDOT was used as a conducting polymer to improve electrode’s sensitivity and stability [14, 27]. Figure 4A shows the improved potential profile of using PEDOT sensor regarding linearity range and stability in background buffer compared to the PEDOT-free electrode. Moreover, casting this hydrophobic conductive layer between the membrane and MWCNTs surface played an important role in reduction of water layer formation and hence minimizing the signal drift. This was proved by applying the water layer test [47] in which relatively stable response to ERY was observed in PEDOT based sensor. Upon switching to interfering ion solution, the potential drift in PEDOT electrode was less significant compared to the PEDOT-free one. The disturbance in potential readings is due to the replacement of ERY by azithromycin in the water film formed between the PVC sensing membrane and the MWCNTs layer. In addition, PEDOT sensor responses showed gradual rise upon re-immersing in ERY standard solution whereas PEDOT sensor exhibited almost null potential drift (Fig. 4B).

The outcome of pH fluctuation on potentiometric readings was investigated through using 1 × 10^−4^ and 1 × 10^−5^ M ERY solutions (Fig. 4C). The figure shows a steady response over the pH range of 4–7. Beyond pH 7, the potential declined significantly due to the gradual decrease in solubility. The influence of different MIP amounts in the ion sensing membrane was studied around the nominal amount (10 mg). Membranes containing lower levels (1 and 5 mg) and higher levels (15 and 20 mg) MIP showed no substantial enhancement regarding slope and linear range. It was also observed that the membrane uniform distribution and homogeneity were impeded by incorporating more than 15 mg MIP. A typical calibration plot for the studied monovalent cation was obtained (Fig. 4D). The linear response was obtained at the concentration range of 1 × 10^−7^–1 × 10^−3^ M ERY with a slope of 51 mV/concentration decade. The electrochemical behavior of the optimized all-solid-state device was assessed using IUPAC standards [25] (Table 2). The collected data during a 4-week period were systematically evaluated. For day-to-day measurements, the sensor provided consistent potential readings. The actual change in the Nernstian slope was roughly 3mV/decade during a 28-day period. The detection limit was calculated using the IUPAC standard and it was found to be 6.6 × 10^−8^ M which is approaching the reported the maximum residue limit value of ERY.

**Table 2:** Electrochemical response characteristics of the proposed chip device

Parameters	Proposed device
Slope (mV/decade)^a^	51 ± 2
Intercept (mV)^a^	259 ± 13
LOD (M)^b^	6.6 × 10^−8^
Response time (s)	6
Working pH range	4–7
Concentration range (M)	1 × 10^−7^–1 × 10^−3^
Stability (Days)	28
Accuracy^a^	99.9% ± 2.6
Correlation coefficient (r)	0.9916
Repeatability^c^	1.6%
Inter-day precision^c^	2.1%
Reproducibility^d^	5.0%

^a^Mean ± SD of five determinations

^b^Limit of detection (measured by interception of the extrapolated arms of calibration plots)

^c^RSD% of nine determinations of 3 ERY concentrations (10^−7^, 10^−5^, and 10^−3^ M)

^d^RSD% of recoveries for the determination of 3 ERY concentrations (10^−7^, 10^−5^, and 10^−3^ M) using two different reference electrodes; a conventional Ag/AgCl double junction (Thermo Scientific, USA) and the miniaturized one

### Selectivity study

One of the most essential properties of potentiometric devices is selectivity, which shows the sensor’s ability to respond to the target analyte over other interfering ions in the solution. The effect of veterinary antibiotics that may be co-administered with ERY and excreted in milk in addition to the effect of inorganic ions (Na^+^, K^+^, Ca^2+^, and Mg^2+^) that are naturally present in the milk matrix was investigated. Ciprofloxacin, tetracycline, and azithromycin were selected as examples of fluoroquinolones class, tetracyclines class, and structurally related compound, respectively. Other antibiotics classes were excluded owing to the absence of positively charged ionizable groups in their chemical structures at the selected pH, and hence not interfering with ERY measurement. The results showed higher selectivity of the MIP-comprised membrane, in the presence of ciprofloxacin and tetracycline, than the NIP-comprised or MIP-free ones (about ten folds enhancement) which is due to the difference in structure and functional groups of ERY from those drugs. The higher lipophilic nature of ERY (Log *P* ≈ 2.6) compared to ciprofloxacin (Log *P* ≈ 1.5) and tetracycline (Log *P* ≈ − 0.8) is also responsible for this good selectivity towards our drug of interest. However, the three comparable membranes showed nearly the same results towards the investigated inorganic ions which is attributed to their limited ion exchange property in the lipophilic membrane. For a further investigation of MIP’s selectivity, another antibiotic with comparable chemical structure was used as a competitive molecule, azithromycin. Despite its higher interfering effect than other antibiotics, the MIP-comprised membrane still revealed better results when compared with the other two membranes. This superiority of MIP-comprised membrane may be attributed to the presence of a distinct nitrogen atom in the macrocyclic ring of azithromycin which does not present in ERY ring [54] (Table 3).

**Table 3:** Potentiometric selectivity coefficients^a^ of the investigated three sensing membranes; MIP-free, NIP-comprised and MIP-comprised ones

Interferent	MIP-free	NIP-comprised	MIP-comprised
Na^+^	1.58 × 10^−3^	1.93 × 10^−3^	1.87 × 10^−3^
K^+^	1.42 × 10^−3^	1.54 × 10^−3^	1.27 × 10^−3^
Ca^2+^	1.28 × 10^−3^	2.34 × 10^−3^	2.03 × 10^−3^
Mg^2+^	2.97 × 10^−3^	2.46 × 10^−3^	1.98 × 10^−3^
Ciprofloxacin	9.50 × 10^−2^	9.69 × 10^−2^	9.34 × 10^−3^
Tetracycline	4.77 × 10^−2^	4.94 × 10^−2^	6.26 × 10^−3^
Azithromycin	8.87 × 10^−1^	6.55 × 10^−1^	9.93 × 10^−2^

^a^Average of three determinations

### Application of proposed device on milk samples

The presented electrochemical chip device was tested in spiked milk samples. The before-mentioned procedures, under Milk samples pretreatment and analysis, were followed, and 50 µL aliquots were then introduced into the chip sample zone. Various ERY concentrations were spiked into the milk samples, and they were analyzed in triplicate. The tabulated results are the mean of three measurements. As shown in Table S2, supplementary information, the results reveal a recovery range of 89.1–98.7%. This reflects the good ability of MIP-comprised sensor to determine ERY in milk samples. Moreover, the results of the highest concentration level (98.7 ± 1.8, *n* = 3) were statistically compared to a reported HPLC method [8] (101.7 ± 1.8, *n* = 3). The calculated *t* and *F* values were 2.74 and 4.23, respectively, and no significant difference was obtained at *p* $\le 0.05$.

### Comparison with previously reported methods

The proposed all-solid-state potentiometric device was compared to other reported electrochemical sensors utilizing MIP for ERY detection. As shown in Table 4, the proposed electrochemical device was the first one employing the simpler and more economic potentiometric technique for ERY determination in milk. As a result, the oxidizable/reducible milk components show insignificant interference. The voltammetric [39] sensor showed lowermost detection limit of ERY in water sample. This might be attributed to lower interferences in such application. Concerning dairy applications, a comparable lowest concentration is achieved by our proposed device. It is worth noting that miniaturization, adopted in this work, allows the use of small sample volumes.

**Table 4:** An overview on reported electrochemical methods utilizing MIP for the determination of ERY

Ref. No	Technique	Scale	Linearity range	LOD	Application
11	Voltammetry	Macro-electrodes	5 × 10^−8^–1 × 10^−7^ M	1.9 × 10^−8^ M	Honey and dairy products
12	Voltammetry	Macro-electrodes	7 × 10^−8^–9 × 10^−4^ M	2.3 × 10^−8^ M	Honey and milk samples
39	Voltammetry	Micro-electrodes	2 × 10^−9^–1.6 × 10^−8^ M	1.2 × 10^−10^ M	Tap water
This work	Potentiometry	Micro-electrodes	1 × 10^−7^–1 × 10^−3^ M	6.6 × 10^−8^ M	Milk samples

## Conclusion

Employing molecular imprinted polymer with miniaturized PCB-based potentiometric sensor improved selectivity, sensitivity, and ease-of-use of erythromycin residue detection in milk samples. The molecular imprinted polymer produced by bulk polymerization technique showed high recognition ability to the analyte in the complex nature of milk matrix. Lower binding capacities were obtained for various classes of antibiotics, such as amphenicols, beta-lactams, fluoroquinolones, sulfonamides, tetracyclines, and a structurally related compound “azithromycin,” compared to erythromycin. The polymer capability was identified and assessed by the aid of different techniques. The designed all-solid-state chip showed high constancy and robustness over a wide concentration range of ERY, thanks to PEDOT conducting polymer. Low potential drifts were obtained in water layer test applied on the proposed miniaturized two electrode system. Although low concentration levels of ERY can be detected by the proposed PCB sensor, future work is still required to improve the detection limit to approach the maximum residue limit of ERY in milk. In conclusion, the proposed PCB-based potentiometric chip utilizes small sample volume besides its feasibility and affordability for end-users. It is evident that this device is a suitable commercial tool for erythromycin detection in dairy samples.

### Supplementary Information

Below is the link to the electronic supplementary material.

Supplementary file1 (DOCX 525 KB)
