# Supplementary material for: All-solid-state chip utilizing molecular imprinted polymer for erythromycin detection in milk samples: Printed circuit board-based potentiometric system
Source: Mikrochim Acta. 2023 Sep 21;190(10):408. doi: 10.1007/s00604-023-05959-w (PMC10514120; doi:10.1007/s00604-023-05959-w)
Supplement: Supplementary file 1 — Supplementary file1 (DOCX 525 KB) [file 604_2023_5959_MOESM1_ESM.docx]

**Supplementary Information**

for

**All-solid-state chip utilizing molecular imprinted polymer for erythromycin detection in milk samples: Printed circuit board-based potentiometric system**

*Mahmoud A. Tantawy *^a,b^, Ali M. Yehia ^a,c^, Heba T. Elbalkiny ^d^*

^a^ Pharmaceutical Analytical Chemistry Department, Faculty of Pharmacy, Cairo University, El-Kasr-El Aini St, 11562, Cairo, Egypt

^b^ Chemistry Department, Faculty of Pharmacy, October 6 University, 6 October City, Giza, Egypt

^c^ School of Life and Medical Sciences, University of Hertfordshire Hosted by Global Academic Foundation, New Capital, Garden City, Cairo R5 New, Egypt

^d^ October University for Modern Sciences and Arts, Faculty of Pharmacy, Analytical Chemistry Department, 11787 6th October City, Egypt

**Corresponding author, e-mail:* [*mahmoud.eltantawy@pharma.cu.edu.eg*](mailto:mahmoud.eltantawy@pharma.cu.edu.eg)*;* [*matantawy@hotmail.com*](mailto:matantawy@hotmail.com)


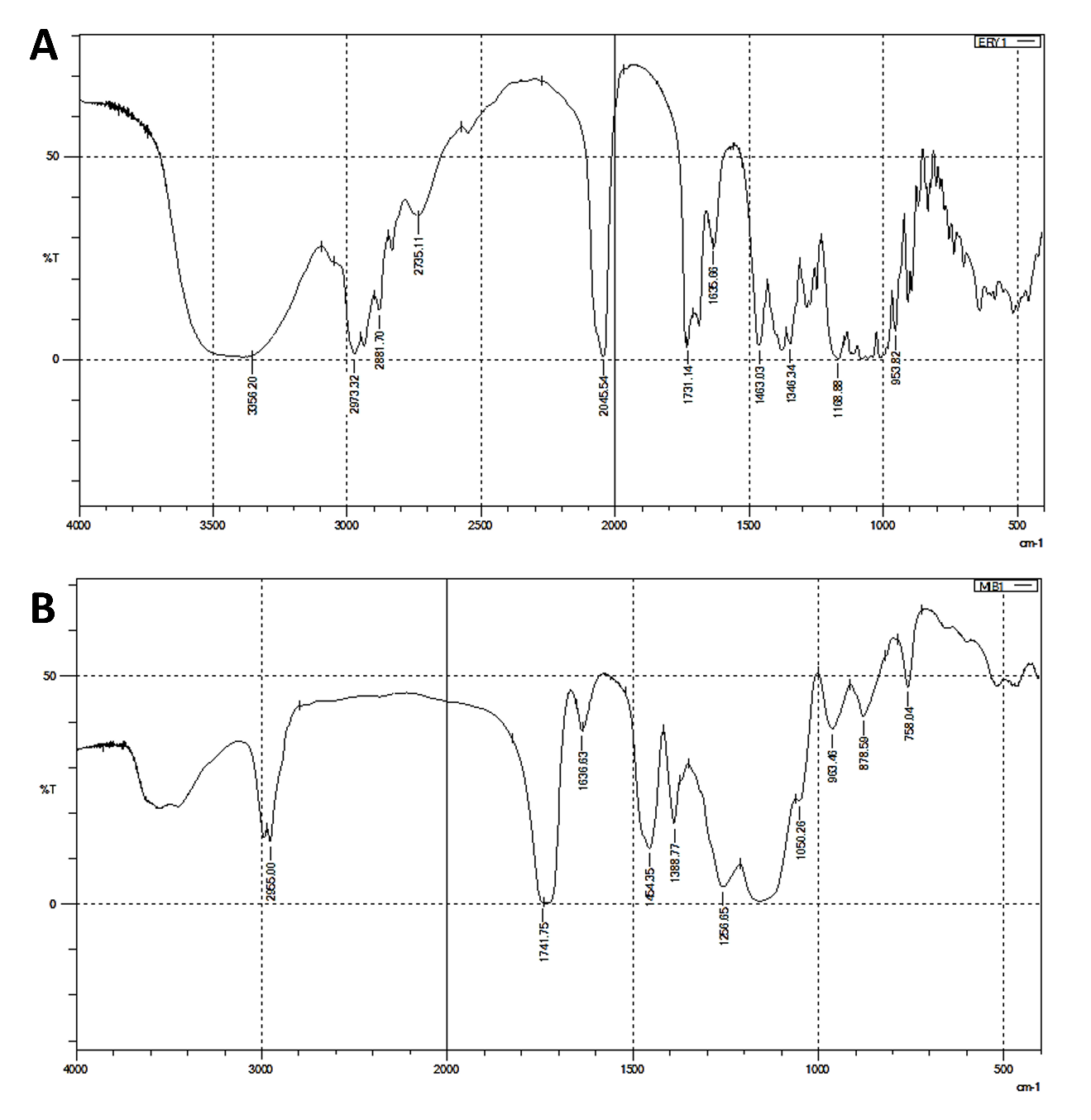


**Fig. S1**. Infra-red spectra of unleached (A) and leached (B) molecular imprinted polymer for erythromycin.

**Table S1.** Results of BET/BJH analysis along with the binding capacity for the prepared polymers; MIP and NIP.

| Polymer | Specific surface area (m^2^ g^-1^) | Pore Volume  (cm^3^ g^-1^) | Average pore diameter (nm) | Q (µmol g^-1^)^a^ |
| --- | --- | --- | --- | --- |
| MIP | 405 | 1.35 | 5.62 | 12.2 ± 0.3 |
| NIP | 312 | 1.24 | 3.78 | 3.6 ± 0.2 |

^a^ Mean ± SD of three determinations.

*

*

**Fig. S2**. Chemical structure erythromycin.

**Table S2**. Application of the MIP-comprised chip device to the determination of ERY in spiked milk samples.

| Sample No. | Added concentration (µg mL^-1^) | Found concentration (µg mL^-1^)^a^ | Recovery %^a^ |
| --- | --- | --- | --- |
| 1 | 1.10 | 0.980 ± 0.032 | 89.1 ± 2.9 |
| 2 | 3.70 | 3.44 ± 0.08 | 93.0 ± 2.0 |
| 3 | 18.3 | 17.4 ± 0.5 | 95.0 ± 2.6 |
| 4 | 36.7 | 36.2 ± 0.6 | 98.7 ± 1.8^b^ |

^a^ Mean ± SD of three determinations.

^b^ Results were statistically compared with that obtained upon applying the reported HPLC method [8]; using Waters XBridge^®^ C18 (250 × 4.6 mm, 5 µm) column with a gradient program of phosphate buffer, pH 7 and acetonitrile as follows: 50% acetonitrile (3 min) at 1 mL min^-1^ and 58% acetonitrile (8 min) at 1.2 mL min^-1^. UV detection at 210 nm.
